# Can families help veterans get more from PTSD treatment? A randomized clinical trial examining Prolonged Exposure with and without family involvement

**DOI:** 10.1186/s13063-022-06183-2

**Published:** 2022-03-30

**Authors:** Laura A. Meis, Shirley M. Glynn, Michele R. Spoont, Shannon M. Kehle-Forbes, David Nelson, Carl E. Isenhart, Afsoon Eftekhari, Princess E. Ackland, Erin B. Linden, Robert J. Orazem, Andrea Cutting, Emily M. Hagel Campbell, Millie C. Astin, Katherine E. Porter, Erin Smith, Christopher D. Chuick, Kristen E. Lamp, Tessa C. Vuper, Taylor A. Oakley, Lila B. Khan, Sally K. Keckeisen, Melissa A. Polusny

**Affiliations:** 1 Center for Care Delivery and Outcomes Research, Minneapolis Veterans Affairs Health Care System, Minnesota, USA; 2 Department of Medicine, University of Minnesota Medical School, Minnesota, USA; 3 VA Greater Los Angeles Healthcare System, Los Angeles, USA; 4 Department of Psychiatry & Biobehavioral Science, University of California, Los Angeles, USA; 5 Department of Psychiatry, University of Minnesota Medical School, Minnesota, USA; 6 Pacific Islands Division, National Center for PTSD, Honolulu, USA; 7 Women’s Division, National Center for PTSD, Honolulu, USA; 8 VA Desert Pacific Healthcare Network (VISN 22), Long Beach, USA; 9 National Center for PTSD, Dissemination and Training Division, VA Palo Alto Health Care System, Livermore, USA; 10 Atlanta VA Health Care System, Decatur, USA; 11 Department of Psychiatry and Behavioral Sciences, Emory University Medical Center, Atlanta, USA; 12 VA Ann Arbor Healthcare System, Ann Arbor, USA; 13 Department of Psychiatry, University of Michigan, Ann Arbor, USA; 14 Minneapolis Veterans Affairs Health Care System, Minneapolis, USA; 15 Department of Psychology, University of Missouri-Kansas City, Kansas City, USA; 16 Department of Family Social Science, University of Minnesota, Minneapolis, USA; 17 Department of Psychology, Augsburg University, Minneapolis, USA

**Keywords:** PTSD, Adherence, Evidence-based treatments, Couples, Family

## Abstract

**Background:**

Posttraumatic stress disorder occurs in as many as one in five combat veterans and is associated with a host of negative, long-term consequences to the individual, their families, and society at large. Trauma-focused treatments, such as Prolonged Exposure, result in clinically significant symptom relief for many. Adherence to these treatments (i.e., session attendance and homework compliance) is vital to ensuring recovery but can be challenging for patients. Engaging families in veterans’ treatment could prove to be an effective strategy for promoting treatment adherence while also addressing long-standing calls for better family inclusion in treatment for posttraumatic stress disorder. This paper describes the methods of a pragmatic randomized controlled trial designed to evaluate if family inclusion in Prolonged Exposure can improve treatment adherence.

**Methods:**

One hundred fifty-six veterans, with clinically significant symptoms of posttraumatic stress disorder, will be randomized to receive either standard Prolonged Exposure or Prolonged Exposure enhanced through family inclusion (Family-Supported Prolonged Exposure) across three different VA facilities. Our primary outcomes are session attendance and homework compliance. Secondary outcomes include posttraumatic stress disorder symptom severity, depression, quality of life, and relationship functioning. The study includes a concurrent process evaluation to identify potential implementation facilitators and barriers to family involvement in Prolonged Exposure within VA.

**Discussion:**

While the importance of family involvement in posttraumatic stress disorder treatment is non-controversial, there is no evidence base supporting best practices on how to integrate families into PE or any other individually focused trauma-focused treatments for posttraumatic stress disorder. This study is an important step in addressing this gap, contributing to the literature for both retention and family involvement in trauma-focused treatments.

**Trial registration:**

ClinicalTrials.govNCT03256227. Registered on August 21, 2017.

## Administrative information

**Table:** 

Title {1}	Can Families Help Veterans Get More from PTSD Treatment? A Randomized Clinical Trial Examining Prolonged Exposure with and without Family Involvement
Trial registration {2a and 2b}.	ClinicalTrials.gov Identifier: NCT03256227; Registered August 21, 2017
Protocol version {3}	September 7, 2021: Version 16.
Funding {4}	This research was supported by a grant from the Department of Veterans Affairs (HSR&D IIR 15-322). The material is the result of work supported with the use of resources and facilities at the Minneapolis Veterans Affairs Health Care System, VA Ann Arbor Healthcare System, and the Atlanta Veterans Affairs Medical Center
Author details {5a}	Laura A. Meis,^1,2^ Shirley M. Glynn,^3,4^ Michele R. Spoont,^1,5,6^ Shannon M. Kehle-Forbes,^1,2,7^ David Nelson,^1,2^ Carl E Isenhart,^8^ Afsoon Eftekhari,^9^ Princess E. Ackland,^1,2^ Erin B. Linden,^1^ Robert J. Orazem,^1^ Andrea Cutting,^1^ Emily M. Hagel Campbell,^1^ Millie C. Astin,^10,11^ Katherine E. Porter,^12,13^ Erin Smith,^12,13^ Christopher D. Chuick,^14^ Kristen E. Lamp,^10^ Tessa C Vuper,^12^ Taylor A Oakley,^15^ Lila B. Khan,^16^ Sally K. Keckeisen,^17^ & Melissa A. Polusny^1,3^ ^1^Center for Care Delivery and Outcomes Research, Minneapolis Veterans Affairs Health Care System ^2^University of Minnesota Medical School, Department of Medicine ^3^VA Greater Los Angeles Healthcare System ^4^Department of Psychiatry & Biobehavioral Science, University of California, Los Angeles ^5^University of Minnesota Medical School, Department of Psychiatry ^6^Pacific Islands Division, National Center for PTSD ^7^Women’s Division, National Center for PTSD ^8^VA Desert Pacific Healthcare Network (VISN 22) ^9^National Center for PTSD, Dissemination and Training Division, VA Palo Alto Health Care System ^10^Atlanta VA Health Care System ^11^Department of Psychiatry and Behavioral Sciences, Emory University Medical Center ^12^VA Ann Arbor Healthcare System ^13^Department of Psychiatry, University of Michigan ^14^Minneapolis Veterans Affairs Health Care System ^15^Department of Psychology, University of Missouri-Kansas City ^16^Department of Family Social Science, University of Minnesota ^17^Department of Psychology, Augsburg University
Name and contact information for the trial sponsor {5b}	Health Services Research & Development, **Robert William O'Brien, PhD, MA,** Scientific Portfolio ManagerHealth Services Research and Development (10X2H)Email: robert.obrien7@va.gov
Role of sponsor {5c}	The sponsor has no role in the design of the study or the writing of the manuscript, nor do they have a role in the collection, analysis, or interpretation of the data

## Introduction

### Background and rationale {6a}

Trauma-focused treatments (TFTs), such as PE, result in significant symptom relief for many patients with PTSD [1, 2]. However, these therapies appear less effective for military personnel and veterans [3]. Multiple meta-analyses link higher dropout rates to attenuated effect sizes across randomized controlled trials of TFTs [4, 5]. Consequently, efforts to optimize treatment adherence may hold promise as a pathway for maximizing the gains veterans can achieve from these treatments. Interventions to improve veteran adherence in real-world delivery of these interventions are particularly warranted, with recent findings demonstrating rates of TFT dropout exceed 60% in national VA clinic samples [6, 7].

Enhanced family support for PTSD treatment may provide one novel approach to improving adherence. Many veterans are highly motivated to involve their loved ones in their PTSD care [8]. Family-involved psychotherapies result in comparable or better outcomes than patient-only treatments for numerous mental health conditions [9–11]. Two couple therapies for PTSD (Cognitive Behavioral Conjoint Therapy and Structured Approach Therapy) have also demonstrated superior effectiveness in reducing PTSD symptoms, compared to either waitlist or family psychoeducation for PTSD [12, 13].

Treatment adherence is infrequently a target of family-involved interventions. However, in a recently completed small RCT (*n* = 40), Thompson-Hollands and colleagues [14] found that a two-session adjunctive intervention with family members, as veterans began PE or Cognitive Processing Therapy (CPT), reduced treatment dropout by 20%, compared to standard PE or Cognitive Processing Therapy. Additionally, Meis and colleagues [15] found that when veterans entering either PE or CPT were encouraged by a close loved one to face things that made them anxious and uncomfortable, they were more than twice as likely to finish one of these TFTs. PE training recommends encouraging patients to talk to their families about their treatment and share treatment materials with them. However, these suggestions are untested, informal, and optional. We are evaluating a systematic method of family inclusion in PE, specifically designed to increase treatment engagement.

This randomized controlled trial (RCT) compares PE as delivered in routine VA clinical care to PE with a family member attending the initial sessions (Family-Supported PE). Family is defined broadly to include intimate partners, blood relatives, and friends. The intervention is designed to help families support veterans during treatment and cultivate a home or social environment that sustains PE participation.

### Objectives {7}

We anticipate that (1) veterans randomized to Family-Supported PE will attend more sessions and report greater homework compliance than veterans randomized to standard PE, and (2) Family-Supported PE will be more effective than standard PE in reducing PTSD severity and comorbid problems. Additional aims include examining barriers and facilitators to implementing Family-Supported PE and evaluating mechanisms driving adherence differences between conditions.

### Trial design {8}

The study employs a two-group (standard PE vs Family-Supported PE), three-site, pragmatic, superiority RCT, using a Type 1 Hybrid effectiveness-implementation design [16]. A total of 156 veteran-family member dyads (312 participants) will be randomly assigned to either standard PE (78 dyads) or Family-Supported PE (78 dyads). This study was reviewed and approved by the VA Central Institutional Review Board. Assessments are administered before treatment, during treatment (self-reports only), posttreatment, and 3 months after treatment (self-reports only).

Practical RCTs use conditions mirroring clinical practice and are intended to directly inform care with generalizable answers to important but simple questions [17]. They rely on randomization and include limited use of elaborate quality assurances, as these efforts are cannot be sustained in clinical care [17–19]. A Hybrid 1 trial design tests an intervention while assessing its potential for real-world implementation [16]. We will examine barriers/facilitators of implementing Family-Supported PE using a mixed-method, multi-stakeholder process evaluation [20] with patients, providers, and mental health leadership. The RE-AIM framework guides interview questions and assessments to evaluate Reach (factors influencing participation rate within target population), Effectiveness (impact of an intervention), Adoption (factors influencing if/how many VA hospitals would adopt the program), Implementation (factors influencing intervention integrity in real-world settings), and Maintenance (sustainability, [21]). Lastly, we collect self-report data from veterans and family members during treatment to explore potential mechanisms generating adherence differences between treatment conditions.

## Methods: participants, interventions and outcomes

### Study setting {9}

Participants are male and female veterans with PTSD symptoms and family members of their choice. Family members include anyone the veteran is close to with whom they have contact at least 3 times a week (referred to hereafter as a support person, SP). All veterans are enrolled in care at one of three participating VA healthcare systems in the USA: VA Ann Arbor Healthcare System, Atlanta VA Health Care System, and the Minneapolis VA Healthcare System.

### Eligibility criteria {10}

Inclusion/exclusion criteria reflect those used in the delivery of PE in routine care, with additional minimal criteria to ensure the dyad can participate safely and effectively. Veteran inclusion criteria involve the following: over 18 years old, enrolled in VA care at a study site, clinically significant symptoms of PTSD, willing to participate with a SP of their choice over the age of 18 with whom they have contact at least 3 times per week, and willing to have sessions and study assessments audio-recorded. Clinically significant symptoms of PTSD are defined as meeting diagnostic criteria for PTSD [22] or subthreshold PTSD on the Clinician-Administered PTSD Scale for DSM-5 (CAPS-5; [23]). Subthreshold PTSD includes endorsement of criteria A (trauma), F (duration), and G (impairment) with at least one symptom from each of the remaining diagnostic criteria [24]. Including veterans with subclinical symptoms is consistent with TFT delivery in routine VA care.

Veteran exclusion criteria include significant suicidal/homicidal ideation requiring primary clinical focus, an episode of mania/psychosis in the past 3 months, diagnosis of Severe Substance Use Disorder in the past 3 months, significant violence in the relationship with the dyad member, anticipated barriers to weekly therapy participation (e.g., major surgery), or failure to complete the baseline survey. SPs with significant symptoms of PTSD or failing to complete baseline surveys are also excluded. Mania, psychosis, and substance use disorders are evaluated using the SCID-5-CT [25]. Suicidal/homicidal ideation are evaluated during a structured clinical interview and through electronic medial chart review. The relationship violence exclusion is defined as an endorsement of severe relationship violence in the past 12 months or current fear of violence in their relationship. Significant symptoms of PTSD for SPs is defined as endorsement of at least one Criterion B item (questions 1–5), one Criterion C item (questions 6–7), two Criterion D items (questions 8–14), and two Criterion E items (questions 15–20) at a severity rating of “moderately” or higher on the PTSD Checklist (PCL-5, 26). With experience with this protocol, we added an exclusion that excluded veterans with a psychiatric hospitalization in either the last 3 months and/or multiple hospitalizations in the past year, to ensure veterans were sufficiently psychiatrically stable to participate.

### Who will take informed consent? {26a}

Participants (both veterans and their SPs) receive an initial study screening over the phone by trained study staff and are provided an information sheet about the study by mail, including all elements necessary for informed consent. When initial screening indicates eligibility, the study staff member reviews the information sheet with the individual. If the individual remains interested, verbal consent is obtained over the phone.

### Additional consent provisions for collection and use of participant data and biological specimens {26b}

This trial does not involve collecting biological specimens for storage

## Interventions

### Explanation for the choice of comparators {6b}

Our primary aim is to determine if integrating family members into PE can improve engagement and outcomes for veterans receiving PE in routine VA care. Consequently, comparing Family-Supported PE to standard PE is the most logical comparison.

### Intervention description {11a}

#### PE

PE is based on Emotional Processing Theory, which posits that PTSD is maintained by fear structures with stimuli, responses, and meaning elements that are excessive and pathological [26, 27]. PE aims to modify the pathological components of these fear structures through activating the fear structures through contact with feared stimuli (e.g., crowds, the memory itself) and experiencing information incompatible with the problematic components of this fear structure (e.g., being surrounded by people is highly dangerous). The primary components of PE are psychoeducation, in vivo exposure which consists of approaching trauma reminders in real life [28] and imaginal exposure which includes repeatedly imagining and describing a trauma memory to the provider. Participants are asked to continue exposure work at home between sessions throughout treatment through in vivo exposure exercises and listening to audio recordings of their imaginal exposure. PE training encourages providers to ask patients to share their educational handouts with their SPs. In keeping with a pragmatic trial, this is not discouraged.

Therapy can be delivered in-person or remotely through video teleconferencing as multiple trials support the effectiveness of both modalities [29–31] and both approaches are widely used within VA [32]. Treatment is delivered with a flexible end date, extending the therapy as needed, resulting in a range of 8 to 15 sessions. Due to the volume of material provided in session 2, VA providers frequently divide Session 2 into two sessions: 2a and 2b. This division also allows for content to be added in 2a and 2b for Family-Supported PE, without requiring more sessions for Family-Supported PE than standard PE. Consistent with how PE is delivered in real-world care, under certain circumstances, providers can conduct off-protocol or “stressor” sessions. These sessions occur in response to patient emergencies, patient crises, or patient refusal to proceed. Family attendance is not explicitly solicited by providers in standard PE. If a SP spontaneously contacts a provider, the provider proceeds as they would in their routine practice, consistent with our emphasis on real-world care.

#### Family-Supported PE

The goal of family involvement in Family-Supported PE is to optimize the veteran’s engagement in PE and teach the dyad to collaborate as a team during treatment. The SP plays the role of a teammate in the veteran’s treatment, like a coach or “workout buddy,” supporting the veteran as treatment progresses. This role is consistent with what is typically referred to as “partner-assisted” or “spouse-assisted” therapy (e.g., [11] [33],). During treatment, the dyad decides together how the SP can be helpful and supportive in treatment, ranging from broad emotional support to practical support for treatment activities (i.e., childcare during imaginal exposure listening exercises at home), to attending in vivo exercises for support and encouragement.

SPs attend the first three sessions (sessions 1, 2a, and 2b). As in the standard PE protocol, these first three sessions focus on education, treatment rationale, and assessment. To build in time for new material, sessions 1, 2a, and 2b are expanded from 1.5 to 2-h sessions. In sessions 4 and 6, the SP joins in person or by phone to share how treatment has been going and troubleshoot any problems with implementing treatment activities at home. SPs are also invited to join the final session of PE to review treatment gains and discuss any next steps for the veteran.

The goals of the adaptations made to treatment for Family-Supported PE are threefold. The first is to build motivation in both dyad members for PE participation. Over the first two sessions, providers complete a case formulation focused on the motivators for and barriers against treatment participation. This formulation helps guide the provider’s discussions with the dyad around treatment commitment and self-efficacy. Providers are instructed to evaluate and optimize the team’s perceptions of PE’s credibility, their self-efficacy for treatment participation, and their commitment to effective participation in PE. Techniques from Motivational Interviewing [34] are built into sessions 1–2b and encouraged beyond that to address any signs of waning treatment motivation.

Our second goal is to increase the frequency, sincerity, and effectiveness of the team’s efforts to acknowledge and encourage one another in approaching trauma reminders and PTSD, using techniques from Behavioral Couples Therapy for substance use disorders [35]. Basic communication skills are used from BCT, such as active listening and paraphrasing. An adapted version of the “Catch and Tell” activity from BCT is used to encourage dyads to reward each other for “approaching PTSD,” including contacting trauma reminders, participating in PE, and discussing treatment. In this activity, adapted from BCT (re-named “Catch and Share”), the dyad is first asked to notice and record when their PE teammate does something to approach PTSD (session 1). In subsequent sessions, they then share these observations with their teammate to encourage this behavior. The concept of reducing SPs’ symptom accommodation is folded into the discussions on “approaching PTSD” for the dyad. Accommodation includes intentional or unintentional behavior by the SP to encourage trauma-related avoidance and fuel PTSD [36]. Basic information on symptom accommodation is shared with the dyad, followed by a discussion of ways to reduce accommodation that could interfere with the success of the veteran’s PE activities.

The final objective is to increase the frequency and ease with which the veterans and their SPs discuss how PE is going outside of the session. This way, the SP can help identify and problem-solve issues the veteran has with treatment, participants can reward each other’s efforts in treatment, and the dyad can break down avoidance habits in their communication with each other. The dyad is assigned to talk at home about what they learned about avoidance at sessions 1 and 2a to stimulate discussions outside of sessions. In session 2b, the dyad is taught to use a Weekly Check-In to continue their discussions about how PE is going for the veteran in rewarding and encouraging ways. The Weekly Check-In is structured and includes tips for discussing critical information, like the veterans’ successes and challenges with treatment, and for ensuring their interaction is mutually rewarding (e.g., SPs are instructed to avoid advising unless requested to do so). The Check-In ends with the Catch and Share activity learned in prior sessions. Use of the Weekly Check-In is routinely discussed throughout the remainder of the treatment sessions to promote adherence with this activity.

### Criteria for discontinuing or modifying allocated interventions {11b}

Participants can choose to discontinue treatment at any time for any reason as participation is fully voluntary. Consistent with the PE treatment protocol, treatment is delivered with a flexible end date, in collaboration with the veteran, based on their response to treatment. See above.

### Strategies to improve adherence to interventions {11c}

#### Therapist training and supervision

Providers are eligible for study therapist training if they have received PE training from the VA’s national Training Program or equivalent training. This PE training includes case consultation with the achievement of competency on a minimum of 2 cases. To standardize intervention delivery, providers receive a Family-Supported PE treatment manual that includes the goals of family involvement, techniques adapted from other interventions (i.e., MI and BCT; 35,36), and session by session instruction and outlines. See Table 1 for an example of the outline for session 1 of Family-Supported PE. Providers participate in a 2-day training in-person at the Minneapolis VA. The training is conducted by the study’s intervention team, which includes experts in PE, family therapy, and MI. Training is targeted to providers without prior experience working with families. Basic information on working with family dyads is covered, including confidentiality, managing conflict, and assessing and responding to reports of relationship violence. The team’s preliminary data is presented, including themes from qualitative interviews with veterans with poor adherence to TFTs and their therapists. This preliminary data highlights common barriers to adherence and sets up the rationale for integrating Motivational Interviewing strategies. We also present the team’s data on family factors that predict treatment dropout. Lastly, the training covers each of the intervention techniques used, including communication training, MI strategies, and adapted BCT exercises.

**Table 1:** Example Session Outline for Family Supported PE (Session 1)

**Session 1** A. Informed consent (15 min) a. Not couple therapy: Target is trauma-related distress, not family problems b. Structure of teammate attendance (3 sessions, phone calls) c. Confidentiality B. Factors that maintain PTSD and Overview of PE from PE Manual (20 min) a. Notice and reflect ‘approach’ talk (Motivational Interviewing) C. Motivational Interviewing (20 min) a. Importance Ruler: Veteran rates and discusses the importance of doing this treatment with their teammate b. Teammate repeats exercise c. Normalize ambivalence: i. Ask for agreement to talk about it as it occurs ii. Discuss what listener should do when doubt is expressed: listen, remind why doing this, encourage to continue D. Establishing what “teamwork” looks like in Family Supported PE (10 min): See Visual Aid a. What does it mean to do this ‘together’? What do each ‘do’? (team discusses) b. Draw parallel between building approach habits and other habit change (e.g., exercise or eating habits) i. Heaving lifting is between sessions (breaking a daily habit takes daily work) ii. Research shows those who lean into the home practice do best c. Teammate acts a workout buddy and rationale for having a workout buddy– see visual aid i. Initiate conversations about how treatment is going, ii. Encourage veteran to do PE tasks and face fears iii. Express your appreciation and commitment iv. Veteran: Encourages teammate to encourage YOU E. Introduce Step 1 in Catch Your PE Teammate Confronting PTSD Exercise (15 min) a. Rationale for noticing and expressing appreciation for approaching distress & doing PE b. Walk through how to use Catch Your Teammate Home Practice Forms c. Ask each for an example from the past week F. Abbreviated Trauma Interview from PE manual with veteran only (20 min) a. Talk to reporter(s) alone about any IPV reported in prior self-reports G. Breathing Retraining from PE manual with teammate present (10 min) and homework assignment a. Assign PE activities from PE manual b. Assign dyad to discuss together what was learned about avoidance	

Following the didactic training, therapists are closely monitored in their administration of Family-Supported PE with their first two cases, including tape review and feedback using fidelity checklists. Providers then participate in weekly consultation calls throughout the trial, consistent with recommendations for overcoming barriers to family involvement in mental health treatment [37].

##### Therapist fidelity monitoring

Sessions are reviewed and coded for fidelity to both Family-Supported PE and standard PE. For Family-Supported PE, we code 20% of the sessions with Family-Supported PE content (sessions 1, 2a, 2b, 4, and 6), and for fidelity to PE, we code PE delivery across 6 sessions per provider. Providers use an online provider dashboard to record session information, reporting any contact between themselves and family members to evaluate for potential cross-contamination between intervention arms.

### Relevant concomitant care permitted or prohibited during the trial {11d}

Due to the design priority on relevance to real-world clinical care, patients are not restricted from receiving psychiatric medication changes or other forms of psychotherapy during their study participation, except another trauma-focused PTSD treatment.

### Provisions for post-trial care {30}

Due to the pragmatic design, there are no restrictions on post-trial care or ancillary care besides that described above. After treatment completion, participants can continue with mental health care as recommended by their VA treatment team.

### Outcomes {12}

Our primary outcomes examine treatment adherence (session attendance and homework compliance). Secondary outcomes include clinical outcomes (symptom improvement in PTSD and depression), quality of life, and relationship functioning. Clinical outcomes are evaluated through self-report and blinded structured clinical interviews. Our selection of which of these measures will serve as the main outcome for this Aim was guided by methodology for practical RCTs. One of the defining principles of a practical RCT is prioritizing outcomes that are simple, clinically friendly, and typical of everyday good practice [17]. In routine VA care, PTSD symptom change is typically evaluated through repeated administration of the PTSD Checklist for the DSM-5 [38]. The PCL-5 is simpler, quicker, and allows for tracking symptom change during treatment better than a resource-intensive, structured clinical interview of symptoms. Thus, clinical outcomes are primarily evaluated through PCL-5 scores, measured throughout treatment, as this is the most clinically relevant approach. Scores from structured clinical interviews are an important secondary outcome to facilitate comparisons with other RCTs, using the Clinician-Administered PTSD Scale for DSM-5 (CAPS-5, 23). Self-reported clinical outcomes are prioritized over blinded interview assessment as they reflect real-world VA evaluation of PTSD symptoms, a defining principle of pragmatic trials [17].

#### Measures

##### Adherence outcomes

Providers record each session attended by veterans on an online provider dashboard. The dashboard information is used to assess protocol adherence. For our primary outcome measure, we count the number of unique protocol sessions attended. The PE manual recommends up to 15 sessions. Sessions can be repeated, and based on patient progress and success, the protocol can be terminated early upon agreement between the therapist and the patient. Thus, the ultimate number of sessions a treatment completer attends varies. As PE’s flexible end date is consistent with how the protocol is delivered in clinical practice, we do not impose an arbitrary maximum number of sessions on patient attendance. Those with a final session note completed by the therapist are considered to have completed all sessions. For our primary outcome, to treat session attendance uniformly across all treatment completers, we code those who completed treatment as having attended 15 sessions. Additional outcomes will consider the total raw number of sessions attended.

Homework compliance scores are the average of provider ratings on the volume of homework completed for all sessions attended, using an item developed for Exposure and Response Prevention Therapy for Obsessive-Compulsive Disorder (also a cognitive-behavioral exposure therapy, [39], and adapted for trauma-focused PTSD treatment, 15). As this score will use an average of all sessions attended, the variable length of treatment is accounted for in the scoring.

##### Clinical outcomes

PTSD symptom severity is assessed using the PTSD Checklist (PCL-5, 26). The PCL-5 is a 20-item questionnaire rating degree to which symptoms “bothered” the veteran in the previous 30 days. Item responses range from 0 (not at all) to 4 (extremely). The CAPS-5 [23] is used for assessing PTSD symptoms. The instrument is a gold-standard, structured clinical interview for diagnosing PTSD and evaluating PTSD severity. Total symptom severity is calculated by summing the severity ratings (0–4) for each of the 20 symptoms of PTSD. Higher scores indicate greater symptom severity. Additional clinical outcomes include depression severity (Patient Health Questionnaire, [40]), quality of life (World Health Organization - Quality of Life, Brief; WHOQOL, [41]), and relationship functioning between the dyad members participating in the study (Quality of Relationships Inventory, QRI, [42]).

##### Potential mechanisms

We anticipate that Family-Supported PE will yield better adherence and outcomes than standard PE through promoting (1) superior attitudes about TFTs, reflected in the constructs of the Theory of Planned Behavior [43], (2) greater SP support for the veteran’s PE participation, and (3) lower rates of symptom accommodation by SPs. The TPB proposes that behavior change can be predicted through intentions for engaging in the behavior, attitudes about the behavior, perceived behavioral control (which includes the concepts of self-efficacy and readiness, [44]), and subjective norms for the behavior. These are assessed through a suite of scales that adapts the TPB to engagement in TFTs [45]. Scales include dyad member’s attitudes about treatment (i.e., Treatment Makes Sense and Treatment Fits Needs scales), perceived behavioral control over treatment participation (Participation Control), and perceived subjective norms for treatment participation (Subjective Norms). The instrument has established convergent, discriminant, and factor validity [45]. Symptom accommodation is assessed through the Significant Others’ Responses to Trauma Scale (SORTS, 37). SP’s support for the veteran’s treatment participation is assessed using items adapted from existing scales of social control and emotional support [46, 47].

##### Moderators

The effectiveness of SP support in influencing veteran treatment adherence may vary with, or be moderated by, SP’s level of stress, family/relationship functioning, and/or therapeutic alliance. SP stress is assessed using the Perceived Stress Scale [48]. Family functioning is measured using the General Functioning Scale from the Family Assessment Device [49, 50]. Relationship functioning is also measured during treatment using one item assessing relationship conflict, developed for the study, and one item assessing relationship satisfaction from the Couples Satisfaction Index [51]. Therapeutic alliance is assessed with the Session Rating Scale [52].

### Participant timeline {13}

**Fig. 1:**
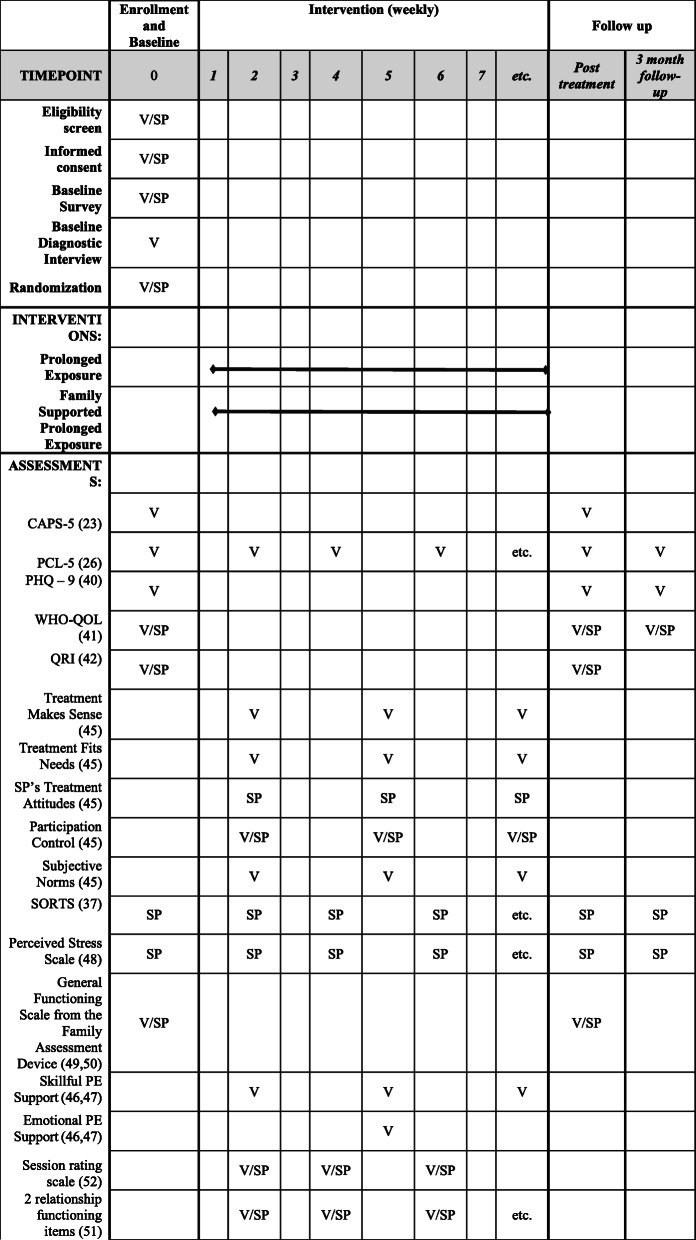
Schedule of enrolment, interventions, and assessments for each dyad member. *Note*. V = Veteran; SP = Support Person. Etc. = pattern of weekly assessments continues in the same pattern throughout treatment. CAPS-5 = Clinician-Administered PTSD Scale for DSM-5; PCL-5 = PTSD Checklist for the DSM-5; PHQ-9 = Patient Health Questionnaire; World Health Organization - Quality of Life, Brief = WHOQOL; Quality of Relationships Inventory = QRI; SORTS = Significant Others’ Responses to Trauma Scale

See Fig. 1 for the intended timeline for each dyad.

### Sample size {14}

The study plans to enroll 156 dyads. Power analyses are based on our preliminary data collected from participants initiating TFTs across four VA hospitals (see 15,45). Veterans attended an average of 7.30 sessions (*SD* = 4.45). Seventy-eight participants in each arm provide 80% power to detect differences between treatments if there is a two-session difference between treatment conditions in the average number of sessions attended. A two-session improvement corresponds to an effect size of (*d* = 0.43), slightly below conventions for a moderate effect size (*d* = .50).

Power analyses for clinical outcomes are based on changes in PCL-5 scores and use estimates of variance and autocorrelation from our prior work (*r* = .61, *SD* = 12.16–14.8). For a plausible range of autocorrelation (*r* = .50–.70) and standard deviations (*SD* = 12–15%), and assuming 20% missingness on posttreatment PCL scores, we should have 80% power or more for underlying differences between interventions of 4–6 points. For a prior version of the PCL [53], a 5-point difference is a minimal threshold for determining if an individual has responded to treatment (i.e., differences not due to chance) and a 10-point difference is the minimal threshold for clinically relevant differences [54]. At the time of planning the study, data establishing a threshold for clinically meaningful differences on the PCL-5 were not available; however, preliminary work suggested the ranges would be similar to those of the original PCL [23].

### Recruitment {15}

#### Veterans

Participants are recruited through multiple avenues, including referral from VA clinic providers, self-referral from veterans responding to study materials, and direct outreach to veterans through mailings and community announcements. Sites are encouraged to promote provider awareness of the study through presentations, attendance at clinical team meetings, and discussions with individual providers. In addition, brochures and flyers are placed throughout the facility in appropriate clinic locations to promote self-referrals. Sites are encouraged to increase awareness of the study in the broader community through networking with local organizations. Direct outreach includes identifying veterans who may be eligible for treatment for a TFT through the electronic medical record (i.e., veterans who received care for PTSD in the past year but were not currently enrolled in a TFT). These individuals are mailed letters introducing the study with instructions for how to opt-out of recruitment efforts. Those who did not directly opt-out are contacted by telephone by study staff.

#### SPs

After obtaining informed consent from veterans, veterans are asked to nominate an SP to participate in the study with them. Similar to veterans, SPs receive mailed letters introducing the study with instructions for how to opt-out of recruitment efforts. Those who did not directly opt-out are contacted by telephone by study staff. Participants originally received a $25 incentive for baseline self-reports, $50 for baseline interviews (veterans only), $50 for posttreatment interviews (Veterans only), $25 for posttreatment self-reports, $40 for 3-month follow-ups, and $5–20 per weekly self-report survey, depending upon the length of the survey. With experience with the protocol, we increased incentives to improve recruitment and retention of participants such that participants receive $40 for baseline self-reports, $50 for baseline interviews (veterans only), $10–20 per weekly self-report survey (depending upon survey length), $50 for posttreatment self-reports, $60 for posttreatment interviews (veterans only), and $60 for 3-month follow-up self-reports.

## Assignment of interventions: allocation

### Sequence generation {16a}

A block randomization schedule is generated that is stratified by a therapist. Dyads are randomly assigned to either standard PE or Family-Supported PE (1:1).

### Concealment mechanism {16b}

The allocation sequence is implemented through the use of a web-based application designed for the study. The sequence is concealed until the project coordinator enters the participants’ final eligibility status and the dyad is ready to be randomized

### Implementation {16c}

Blocks are generated by the statistician and implemented by the study data systems developer using a web-based application. Participant dyads are randomly assigned to an intervention at the level of the veteran-SP dyad when they have completed informed consent and have been evaluated for on all inclusion/exclusion criteria. The web-based application informs the study site’s project coordinator of the dyad’s assignment. The dyad is notified of their assigned intervention when it is time to schedule the first treatment session.

## Assignment of interventions: Blinding

### Who will be blinded {17a}

Assessors and the investigator overseeing assessment training and supervision are blinded to participants’ treatment condition. Blinded staff do not have access to participants’ treatment condition within the study’s web-based application. At baseline assessments and at the completion of the posttreatment clinical interviews, assessors remind participants that they are blinded and request that participants do not disclose their randomization.

### Procedure for unblinding if needed {17b}

We do not anticipate any circumstances under which the assessors would need unblinding.

## Data collection and management

### Plans for assessment and collection of outcomes {18a}

#### Baseline assessments

After providing informed consent by telephone, participants complete baseline surveys by mail, which include a more detailed assessment of family violence and PTSD symptoms. After completing mailed surveys, veterans are scheduled for a structured clinical interview delivered by centralized telephone assessment, described below.

#### Assessments during treatment

During treatment, providers record treatment adherence (i.e., weekly session attendance and homework compliance) using an online provider dashboard. Self-reported PTSD symptom severity and relationship functioning are assessed at baseline, bi-weekly during treatment, posttreatment, and in 3-month follow-up surveys. The remaining clinical outcomes are also measured at baseline, posttreatment, and in 3-month follow-up surveys. PTSD symptom severity is also assessed through blinded structured clinical interviews at pretreatment and posttreatment, discussed below. Measures of potential mechanisms underlying differences between conditions are assessed by both veteran and SP self-report instruments during treatment. See Fig. 1 for a schedule of measures.

#### Posttreatment and 3-month follow-up assessments

Regardless of treatment completion, the study team administers posttreatment and 3-month follow-up assessments. Posttreatment assessments are conducted at treatment termination for those who successfully finish treatment. Those who prematurely discontinue treatment are assessed at 16 weeks post-session 1. For those who never attend a session, assessment occurs at 16 weeks post-randomization. Posttreatment assessments include a structured clinical interview to assess PTSD symptom severity (veterans only), mailed surveys from both veterans and SPs, and a qualitative interview with veterans who participated in Family-Supported PE to identify facilitators of and barriers to implementation of the intervention.

#### Centralized telephone assessment

Veterans complete structured clinical interviews at pre- and posttreatment by telephone from the hub site. The quality and acceptability of phone administration of such interviews are well-established [55, 56]. Phone administration reduces the burden on participants, and centralization reduces variation across sites in fidelity to the assessment [57]. All structured interviews are administered by independent, doctoral-level assessors, who are blinded to the assigned study intervention. The CAPS-5 [23] is administered for symptom severity and minimum symptoms necessary for study inclusion. The Clinical Trials Version of the Structured Clinical Interview for DSM-5 (SCID-5-CT; 25) is used to assesses study exclusions, including mania, psychosis, and substance use disorders.

Interviewer training includes an online CAPS-5 [23] training course developed by the National Center for PTSD, relevant readings (SCID-5-CT manual, 25, CAPS-5 manual, 23), and instruction with a blinded study team member with expertise in the field. Instruction includes a minimum of two practice interviews for each rater, reviewed by the expert, with additional practice interviews as indicated, and supervision throughout the trial. To ensure assessors meet high-quality standards and prevent drift in scoring, periodic calibration exercises are completed. Audio recordings for 10% of administered interviews are reviewed by a second assessor and scored independently. The two assessors and the expert meet to compare scores and discuss reasons for variations in item scores.

## Process evaluation

The barriers and facilitators of real-world implementation of Family-Supported PE are examined through a mixed-method, multi-stakeholder process evaluation with patients, providers, and mental health leadership [20]. The RE-AIM framework guides interview questions, assessing Reach (factors influencing participation rate within target population)*,* Effectiveness (impact of an intervention), Adoption (factors influencing if/how many VA hospitals would adopt the program), Implementation (factors influencing intervention integrity in real-world settings), and Maintenance (sustainability; 21). Data sources include study outcomes described above, a patient screening database, a team observational process log, fidelity monitoring data, post-intervention qualitative interviews with providers and mental health leadership, qualitative exit interviews with veterans in Family-Supported PE, and SP reports on treatment experiences from posttreatment surveys. See Table 2, adapted from Hagedorn and colleagues [20]. The patient screening database reflects the number of participants screened and reasons for exclusion/inclusion. The process log includes observations and reflections on the team’s daily experience with essential activities in implementing Family-Supported PE within the trial. The log records comments from clinical staff and patients not readily reported in formal data collection methods [20]. Fidelity monitoring contributes to the evaluation of training effectiveness, intervention integrity, and which intervention strategies proved particularly difficult/easy to master. Veterans participate in qualitative open-ended exit interviews after treatment completion. Interviews assess reactions to the family-supported intervention, how SPs helped or hindered veteran treatment engagement, and recommendations for intervention changes.

**Table 2:** Implementation Framework Elements Guiding Process Evaluation

Element	Key Question	Data Sources
Reach	• Percentage approached who agree to participate? • Differences between participants and non-participants? • What influences willingness to participate?	• Patient screening database • Process log • Veteran exit interviews • SP self-reports
Effectiveness	• What is the effect of the intervention?	• Study outcomes • Veteran exit interviews • Provider and mental health leadership interviews
Adoption	• Greatest barriers to adoption? • Supports needed for clinics to adopt the intervention?	• Process log • Provider and mental health leadership interviews
Implementation	• What supports are needed to ensure consistent intervention delivery? • What tools are needed for consistent intervention delivery?	• Process log • Provider and mental health leadership interviews fidelity monitoring • Veteran exit interviews • SP self-reports
Maintenance	• What resources are needed to maintain the intervention? • What adaptations are needed to integrate into regular practice?	• Process log • Provider and mental health leadership interviews • Fidelity monitoring

## COVID-19 addendum

The COVID-19 pandemic began part-way through data collection. Providers at each site who were delivering therapy in-person transitioned to remote delivery, primarily via videoconferencing, and occasionally via telephone. Procedures were altered to allow the study to continue. Study exclusion criteria changed to require participants to participate through videoconferencing, to have access to a device with the internet, and to have a private space for therapy sessions. Coordinators and providers worked with participants to problem-solve these issues (e.g., participating from the car or the garage for privacy). The process evaluation was broadened to include questions evaluating the impact of the ongoing and technology on treatment delivery to better contextualize treatment findings. We also began offering survey administration online versus solely by mail or in-person. Online surveys were shorted to optimize response rates by removing measures that were of lesser interest and not required to address the study’s aims (e.g., SP’s PTSD symptom severity, the veteran’s trauma-related cognitions).

### Plans to promote participant retention and complete follow-up {18b}

We use our team’s well-established modified-Dillman protocol [58] with repeated mailings, phone calls, and incentives to optimize response rates (incentive schedule described above; 56). For surveys of Veterans with PTSD, our team’s response rates from prior research studies have ranged from 55 to 85%. We offer telephone administration to optimize response rates and emphasize the distinction between treatment participation and participation in study assessments to encourage those who discontinue treatment to continue to participate in assessments. As our primary outcome is treatment adherence obtained by provider reports, the primary outcome will be available for all participants.

### Data management {19}

All data is stored on the servers for the research center where the hub site is located. We use “standalone,” secure data servers that are accessible only to designated, security-cleared, and trained personnel, and data are de-identified as quickly as is feasible.

#### Maintaining server security

All individuals with administrative access privileges to the center’s servers include IRM personnel and the Statistics and Data Management Team (SDMT). These individuals have been screened and assigned a security clearance putting them in trusted positions of the hospital with clearances to work with patient-level data. These individuals and their access to the center’s servers is ultimately monitored and controlled by a Senior Program Analyst within the SDMT. IRM’s access to the data is strictly limited to backing up server data, which prevents catastrophic loss of data. Backups are written to tapes that are stored in a secure location accessible only to IRM personnel. The SDMT maintains permissions, data storage, and all server applications.

#### Organization and access to research data

Data on the server are organized by projects within folders designated by each investigator. Only members of a given project have access to a specific project folder on the server. Even then, access to project data is obtained through Windows authentication (i.e., user’s name and password to the network). No person without a login name and password to the hospital’s domain network can access data on the center’s servers. Thus, all data housed on the server are extremely secure, and access by unauthorized persons is highly unlikely. Data containing patient identifying information are stored on the servers accessible only to SDMT members who are directly involved in the project. Thus, not even the PI can link individual names to their PHI without first obtaining permission to do so from the SDMT. These protections exceed the usual protections provided for protected health information by the hospital system.

#### Securing confidential research data

Completed surveys are mailed in sealed envelopes using USPS, UPS, or FedEx with a tracking number to the hub site. Extractions of secondary data (i.e., electronic medical record data) are stored on servers accessible to the research center’s SDMT only. Secondary data are de-identified according to HIPAA criteria and provided a random study identification number. A crosswalk table is created linking the study id with the primary key of the data source. Primary data contain only the coded study identification number to identify study participants. The paper version of any forms/surveys is kept in locked cabinets within a locked room. Data from these surveys/forms are scanned or data entered by project staff or the research center’s SDMT members to a secure folder.

Upon receipt, approved study staff upload completed surveys to the secure server where they are accessible to the SDMT. Scanned surveys remain in locked cabinets within a locked room at the site as described. Data collection involving direct data entry is performed through a custom application written by a SDMT programmer. This ensures that data is located in a secure environment and accessible to only those individuals with permission. Only individuals with a need to access the data, as vetted by the project’s Principal Investigator, are granted access. Even then, only the absolute minimum number of data elements is released.

#### Data used for analysis

The final quantitative data will be constructed in Statistical Analysis Software (SAS) data sets. Quantitative analyses are mostly conducted by statisticians assigned to the project or by other members of the project (e.g., Principal Investigator). Qualitative data includes audio recordings of interviews that are transcribed and then stripped of all identifiers. De-identified transcripts are then uploaded into qualitative analysis software (NVIVO). Qualitative analyses are conducted by study investigators with qualitative expertise. SAS data sets and qualitative transcripts will be de-identified according to HIPAA criteria, using only a subject’s coded identification number as the primary key. The de-identified data set will be made accessible to those project members who are conducting analyses. Only the data elements required for the analysis under consideration are released.

### Confidentiality {27}

For all participants, strict confidentiality procedures are maintained to minimize the potential risk of loss of confidentiality. Participant privacy is further assured by conducting interviews in a private office and by assigning arbitrary identifiers in place of individual names in the field notes. Data analysis, interpretation, and reporting are based on these de-identified field notes and transcriptions. Since subject responses are not linked to identifying information, participant confidentiality is assured.

Names, social security numbers, and contact information does not appear on any study materials. Instead, only unique study identification numbers randomly assigned to each record are used as described above. Only site lead investigators, project coordinators, and study programmers (when extracting data to obtain treatment adherence and compare survey responders to non-responders for the survey) have access to an encrypted crosswalk table linking study identification numbers to identifying information. Access to individual project data on the server is granted only to project staff by a SDMT member, as authorized by the study investigator. Identifiers are destroyed as quickly as possible. Audio recordings (i.e., qualitative interviews) are stored digitally on center servers and only accessible to the principal investigator and project coordinator. Participants are asked not to use last names or provide identifying information during recorded interviews.

### Plans for collection, laboratory evaluation, and storage of biological specimens for genetic or molecular analysis in this trial/future use {33}

This is not applicable. No biological specimens for genetic or molecular analysis are used in the current trial.

## Statistical methods

### Statistical methods for primary and secondary outcomes {20a}

#### Adherence and clinical outcomes

Analysis of the adherence outcomes will employ generalized linear mixed models and an intent to treat approach. We will initially consider a linear mixed effects model for the number of unique protocol sessions attended using intervention condition and study site as fixed effects predictors and including random effects for therapists. Alternate models comprising mixed effects Poisson and negative binomial regression models, with log link, will be considered if the linear mixed effects model does not fit the data. A similar approach will be used to analyze the number of sessions attended. Analysis of the proportion of homework completed will initially consider a mixed effects beta regression with logit link, random effects for therapists, and fixed effects for intervention condition, number of sessions completed, and site. Likelihood ratio test methods will be used to test for intervention effects, using a .05 significance level, and intervention effects will be quantified using model-based effect estimates with corresponding confidence intervals.

Analyses of the intervention effects on outcome measures derived from the PCL-5, CAPS-5, PHQ-9, WHOQOL, and QRI scores will use similar mixed effects models with similar likelihood ratio testing methods and model-based summarization of estimated effects. For dichotomous outcomes, such as the presence of PTSD or achieving a clinically significant reduction in CAPS-5, we will employ mixed effects, logistic regression models. For non-binary outcomes, the models will use distribution and link function components that yield well-fitting models for the given outcome.

With a random assignment to intervention, we do not anticipate imbalances in baseline characteristics between groups, but we will evaluate bivariate associations between condition assignment and covariates predictive of outcomes and include any imbalanced covariates in the respective regression models as additional predictors.

### Interim analyses {21b}

No interim analyses are planned.

### Methods for additional analyses (e.g., subgroup analyses) {20b}

#### Potential mechanisms

We will explore indirect pathways from Family-Supported PE to improved treatment outcomes through greater adherence (e.g., treatment condition increases session attendance which leads to a greater reduction in PTSD symptoms). The analytic approach will build off of the models discussed above, with the potential mechanisms incorporated as mediators. Indirect effects for mediators treated as single summary scores will be directly tested using bootstrapped confidence intervals [59] to avoid the often-violated assumptions underlying Sobel’s [60] method.

#### Process evaluation

Quantitative data sources for the process evaluation include study outcomes and patient screening databases. Patient screening databases will be examined descriptively to answer the key questions outlined in Table 2. Qualitative interviews and process log entries will be examined using a rapid turn-around analytic approach for qualitative data [61, 62]. Hamilton’s approach [61, 62] is a team-based method that can be used to obtain rich qualitative results in a brief amount of time. Interviews will be audio-recorded and transcribed. The first step of analysis is data reduction (an analytic approach that sorts, focuses, and organizes data [63];). The qualitative analysis team will draft, field, and revise templated forms for use by staff to summarize each interview transcript. Summary points will be transferred from templates into data matrices that organize points for each RE-AIM factor by population (i.e., veteran, provider, leadership). The qualitative analysis team will meet to provide their impressions of the matrix contents and create a final memo summarizing the findings and key themes that emerged.

### Methods in analysis to handle protocol non-adherence and any statistical methods to handle missing data {20c}

We will have complete data for the adherence measures, but the clinical outcome measures will be subject to attrition and follow-up nonresponse. We will examine associations between intervention, baseline covariates, treatment adherence, and treatment progression with the presence of missing data. We will use the information gained from these analyses to assess the potential impact of missing data on the initial analysis results. We will implement a chained series of regression models to multiply impute the missing data and reimplement the analyses above using the imputed data, aggregating results using standard methods for multiple imputation. In addition, we will incorporate these results in the development and implementation of selection models to assess the potential impact of both missing at random and nonignorable missing data.

### Plans to give access to the full protocol, participant-level data, and statistical code {31c}

There are no plans for granting public access to participant-level data or statistical code. A full version of the study protocol will be available upon reasonable request.

## Oversight and monitoring

### Composition of the coordinating center and trial steering committee {5d}

For this three-site study, the hub site is responsible for coordinating among sites and ensuring consistency across sites in implementing the study protocol. The PI meets at least weekly with the project manager, the site PIs, and site coordinators. In addition, the site coordinators keep frequent, regular contact with the project manager through email, instant messages, phone calls, one-on-one meetings, and group meetings. Study therapists participate in weekly case consultation meetings with a co-investigator responsible for oversight of therapy delivery and/or the PI. Through these and other contacts by email, phone, and ad hoc meetings we (1) ensure adherence to the protocol, (2) keep engaged sites informed of changes to the protocol, informed consent, and HIPAA authorization, (3) inform local sites of any serious adverse events or unanticipated problems that may impact the conduct of the study, (4) ensure that required local site approvals are obtained, and (5) notify all local facility directors and site investigators when the study reaches the point that it no longer requires the engagement of the local facility. The study team also reviews relevant sections of the protocol periodically to ensure all phases of the study are conducted according to the IRB-approved protocol.

### Composition of the data monitoring committee, its role and reporting structure {21a}

We also report at least annually to the HSR&D Data and Safety Monitoring Board (DSMB). The HSR&D DSMB is a national board that monitors enrolment and safety of all multi-site research funded by VA HSR&D. The DSMB reviews the protocol, analysis plans, study progress, recruitment rates, protocol compliance, data collection processes, and adverse events at least annually. DSMB members are appointed by the Director of HSR&D. Voting members include two HSR&D investigators, a health economist, a physician, two biostatisticians, and an expert in human protection issues. Voting members declare conflicts of interest and do not participate in board activities for any project for which they are conflicted. Further details can be found here: HSR&D Data and Safety Monitoring Board (va.gov).

### Adverse event reporting and harms {22}

This project operates under the oversight of the VA Central IRB for Protection of Human Subjects. Further, we log all phone calls received from any participants and carefully evaluate any concerns raised about the protocol. Reporting covers (1) safety of study participants (e.g., unanticipated serious adverse events), (2) study enrollment relative to expectations, (3) characteristics of study participants, and (4) retention of study participants at posttreatment evaluation.

Adverse events (AE) include any untoward medical occurrence in a patient or clinical investigation subject administered an intervention and which does not necessarily have a causal relationship with this treatment. We collect the following safety information (adverse events) that occurs within 7 days leading up to the final assessment: (1) suicide attempts, (2) psychiatric hospitalizations, (3) and episodes of significant family violence. Therapists are instructed to notify study staff immediately when such events occur during treatment delivery. Staff report directly to the local site PI regarding any events. Therapists meet regularly for case consultation with a study investigator, where the occurrence of any of these events is further discussed and tracked. Questions regarding the occurrence of each of these three events are included in posttreatment assessments. Data obtained from participants are reviewed for safety concerns. In the case of problems, the staff discusses this with the appropriate site PI.

Events are immediately communicated to the study PI, who is a licensed clinical psychologist. The site PI, in turn, reports any problems to the central IRB. Once learned of any serious adverse events, unanticipated problems, compliance issues, and/or protocol deviation from the study coordinator or local site PIs, the study reports these events to the IRB within 5 business days of learning of its occurrence. If there are modifications or amendments to the study, protocol amendments are submitted to the IRB and the team awaits approval before implementation.

### Frequency and plans for auditing trial conduct {23}

All processes for oversight and monitoring of trial conduct are described elsewhere in this manuscript. The IRB reviews the conduct of the study annually through their continuing review process.

### Plans for communicating important protocol amendments to relevant parties (e.g., trial participants, ethical committees) {25}

Protocol amendments are submitted to the IRB for review. Any deviations from the protocol will be fully documented using a breach of report form. Important approved amendments are communicated to the DSMB through a templated annual report. Trial participants are notified of any important approved amendments when needed by telephone. The clinical trial register record is updated annually.

### Dissemination plans {31a}

We will publish findings in high-quality health services and clinical research journals, prepare synopses as research briefs for VA and media releases, and present at national meetings and conferences. We will develop an executive summary of findings to disseminate to administrators at the National Center for PTSD, the VA Office of Mental Health and Suicide Prevention (OMHSP), and VA Family Services. Versions of these materials will be developed for providers, veterans, and families. Written materials will be disseminated through the VA websites, intranet sites, list serves, and SharePoint sites.

## Discussion

Family involvement has been highlighted as a fertile avenue for improving PTSD treatment [1, 64]. Supporting this, Laws and colleagues [65] found that even one family-involved psychotherapy visit was associated with improvement in veterans’ PTSD symptoms. Observational research shows close relationships play a critical role in stimulating veterans’ treatment-seeking behavior [66, 67] and family encouragement reduces PTSD treatment dropout [15]. Lastly, Thompson-Hollands and colleagues [14] reduced treatment dropout by 20% when family members were randomized to participate in a two-session adjunctive intervention versus when veterans participated in standard TFT [14].

Two novel psychotherapies that treat PTSD in a conjoint context have established effectiveness compared to waitlist [12] and family psychoeducation [13]. However, as of this writing, there is only one small published randomized controlled trial that explicitly compares a family-integrated TFT to an individual approach to TFT (*N* = 40; 14). While the importance of family involvement in PTSD treatment is non-controversial, a lack of evidence leaves patients and providers without guidance as to which strategies for family involvement (if any) work better than one-on-one approaches to TFTs. Until this evidence-based is established, calls to mainstream family involvement in PTSD treatment [1, 2, 68, 69] will likely remain unanswered. High-quality, clinically relevant, practical clinical trials that establish this evidence base are needed.

Family involvement can be conceptualized as a continuum from a very modest level of conjoint family involvement (e.g., family attendance at a PTSD educational workshop, [70]); brief adjunctive approaches of working separately with SPs [71]; to fully dyadic approaches where a SP participates in every aspect of care (e.g., Cognitive Behavioral Conjoint Therapy, 72; Structural Approach Therapy, 13). Family-Supported PE offers a middle ground in this spectrum of family involvement, by inviting a SP into the first three sessions with the veteran to help the dyad learn skills to work together as a team in PE.

Our choice to compare standard PE to this middle ground in family inclusion was informed by two factors. First, efforts to mainstream family involvement in PTSD treatment are likely more effective if patients have options for involvement that range from the spectrum described above. Promising fully dyadic approaches to treating PTSD have been developed and tested [13, 72]. Comparatively less work has been conducted developing methods of family integration where a family member does not attend every session. Thompson-Hollands and colleagues [73] found that when veterans struggled with TFT adherence, their SPs had little to no knowledge of the treatment, including its basic goals and activities. Through teaching SPs and veterans how to work together to get the most out of the treatment, we hope to help dyads embrace and more actively engage in the treatment, so veterans can get more out of PE than they would have alone. We anticipate these goals are achievable through a modest level of family involvement.

Secondly, many VA mental health providers have limited training and exposure to working with families. Mounting a limited number of conjoint sessions to augment an intervention in which many providers already have the expertise (PE) may be less intimidating for these providers and minimize the intensity of the workforce training initiative. This also means fewer sessions for providers to coordinate attendance for both veterans and their SPs and fewer practical burdens associated with these family sessions. Greater practical burdens with conducting family sessions and provider concerns about their limited skills and confidence in family interventions are two frequently cited barriers to wide-scale use of family-involved psychotherapies [37].

### Strengths and challenges

To the best of our knowledge, we are conducting the largest study to date to test the effectiveness of evidence-based treatment for PTSD with and without family involvement. The study is being conducted across three diverse VA hospital settings and includes both mixed methods and dyadic data (both veteran and SP reports). Consistent with how PE is delivered in VA care, our inclusion/exclusion criteria were intentionally as broad as possible and include veterans with subclinical symptoms.

Two important features of our study design are worthy of additional discussion. One is the integration of a process evaluation into the study design. This process evaluation will identify potential barriers to wider implementation of Family-Supported PE in real-world VA care and possible solutions to these barriers. This can expedite the translation of our findings into routine care. This data will also assist in contributing to the broader literature identifying and exploring barriers to implementing family-involved psychotherapies in clinical practice.

Secondly, we are testing Family-Supported PE using pragmatic design features. For example, the interventions are being delivered (1) under training and supervision conditions intended to mirror real-world care rather than ideal conditions within a tightly regimented efficacy trial, and (2) by existing VA clinicians instead of clinicians interviewed and selected specifically for this trial. The benefit of this approach is that, if differences are found between treatment conditions, findings are more relevant to real-world care. The differences may be more robust to replication in future effectiveness trials and more likely to generalize to routine practice.

These choices come with limitations. For example, if differences are not found between treatment conditions, this may be due to the selection, training, and/or monitoring of providers. We cannot preclude the possibility that (a) more stringent controls over the intervention delivery and/or (b) utilizing therapists with expertise in family interventions and efficacy trials would have yielded a different result. Our process evaluation, including qualitative interviews with providers and veterans, will help us better understand the implications of these choices.

### Conclusions

PTSD is a significant health problem both in the veteran and civilian community. While several evidence-based treatments are available for PTSD, treatment retention remains an issue that negatively impacts treatment outcomes. Family involvement in care has the potential for facilitating successful engagement in evidence-based individual interventions. Results of this trial will contribute to the literature on both PTSD treatment retention and family involvement in TFTs.

## Trial status

As of the date of this writing, the IRB protocol was last revised on September 7, 2021 (Version number 16). Recruitment was open to dyads to serve as therapist’s first training cases on November 1, 2017. RCT recruitment began as therapists completed training cases. The last case was randomized on August 8, 2021. As of this date, 7 dyads are in the active treatment phase of the study. The manuscript was not submitted earlier as (1) manuscript preparation was delayed due to focusing resources on managing the impact of the pandemic on the study and impact of the pandemic on the study team’s time and (2) the manuscript was first submitted to a different journal in the last months of recruitment.

## Abbreviations

CAPS-5: Clinician-Administered PTSD Scale for DSM-5
CPT: Cognitive Processing Therapy
HSR&D: Health Services Research and Development
IRB: Institutional Review Board
MI: Motivational Interviewing
OMHSP: VA Office of Mental Health and Suicide Prevention
PCL: Posttraumatic Stress Disorder Checklist (DSM-IV)
PCL-5: Posttraumatic Stress Disorder Checklist (DSM-5)
PE: Prolonged Exposure
PI: Principal Investigator
PTSD: Posttraumatic stress disorder
QRI: Quality of Relationships Inventory
RCT: Randomized controlled trial
SCID-5-CT: Clinical Trials Version of the Structured Clinical Interview for DSM-5
SDMT: Statistics and Data Management Team
SORTS: Significant Others’ Responses to Trauma Scale
SP: Support person
VA: Veterans Affairs
VISN: Veterans Integrated Service Network
WHOQOL: World Health Organization - Quality of Life, Brief
